# The application of bioglass to treat osteoarthritis

**DOI:** 10.17179/excli2023-6613

**Published:** 2023-12-04

**Authors:** Saurav Kumar Jha, Bhupendra Kumar, Keshav Raj Paudel, Amitabha Bandyopadhyay

**Affiliations:** 1 Department of Biological Sciences and Bioengineering (BSBE), Indian Institute of Technology, Kanpur, 208016, Uttar Pradesh, India; 2 Centre for Inflammation, Faculty of Science, School of Life Science, Centenary Institute and University of Technology Sydney, Sydney, 2007, Australia

## ⁯

The ability of articular cartilage to regenerate is very slow, owing to its limited number of cells and lack of blood supply. Consequently, in cases where cartilage abnormalities remain untreated, there is an increased probability of their degeneration, ultimately resulting in the development of osteoarthritis (OA) (Onishi et al., 2012[7]). The existing treatment strategies for OA mostly focus on mitigating symptoms. Therefore, it is essential to examine several techniques, such as those pertaining to the regeneration and maintenance of cartilage. Although knee replacement surgery may be judged necessary in some cases, it is not recommended for those in younger age groups and is not regarded a feasible long-term strategy for controlling OA, even among older patients (Liu et al., 2021[6]). Bioactive glasses, also referred to as Bioglass, have shown considerable promise as an approach for enhancing bone regeneration when compared to other bioactive ceramics. Nevertheless, it is important to acknowledge that bioactive glasses, despite their favorable characteristics, have not attained comparable levels of commercial success as other bioactive ceramics (Jones, 2013[3]). With respect to other bioceramics, bioactive glasses have been demonstrated to link with bone more quickly in *in vivo* studies. Nevertheless, *in vitro* research has linked the osteogenic potential of bioactive glasses to their dissolution products, which have been shown to stimulate osteoprogenitor cells at the genetic level (Santocildes‐Romero et al., 2015[8]). Remarkably, even after four decades of extensive investigation conducted by several research teams, no alternative bioactive glass composition has been discovered to exhibit superior biological characteristics as compared to the initial Bioglass 45S5 composition. Although, calcium phosphates, namely tricalcium phosphate and synthetic hydroxyapatite, are more often used in clinical settings. There are several factors contributing to this, including both economic considerations and the scientific constraints associated with the original Bioglass 45S5 formulation (Jones, 2013[3]). One of the issues faced in the manufacturing process of porous bioactive glass templates (scaffolds) for bone regeneration with Bioglass 45S5 is the occurrence of crystallization during the procedure of sintering, which presents challenges. But the underlying principle of bone regeneration involves the use of a scaffold capable of serving as a three-dimensional (3-D) provisional framework, facilitating the process of bone restoration. Ideally, the scaffold will elicit the activation of the innate regeneration processes inherent in the human body. Consequently, the scaffold must engage cells, such as stem cells derived from bone marrow, and induce their differentiation into new bone tissue. For the new bone to sustain viability, it is essential that blood vessels successfully infiltrate the area. Over a period, the scaffold is expected to undergo degradation, so allowing for the normal process of bone remodeling to occur. Notably, Lin et al. designed a copper-incorporated bioactive glass-ceramics (Cu-BGC) scaffold, that demonstrated to facilitate the process of cartilage regeneration and the restoration of the osteochondral interface, together with the suppression of inflammatory response. Thus, it exhibits the potential to hinder the progression of OA in individuals with osteochondral abnormalities (Lin et al., 2019[5]). Similarly, Xue et al. investigated the influence of the incorporation of Bioglass into Poly (3-hydroxybutyrate-co-3-hydroxyvalerate) (PHBV) 3-D porous scaffolds on the characteristics of the cartilage progenitor cells (CPCs). In contrast to the pure PHBV scaffold, the PHBV/10% Bioglass scaffold exhibits enhanced hydrophilicity and a greater proportion of attached cells. The CPC-PHBV/10% Bioglass built had superior outcomes in terms of cartilage-like tissue formation, exhibiting elevated expression of genes associated with cartilage and increased synthesis of cartilage matrix proteins. Additionally, this construct exhibited enhanced biomechanical performance compared to the pure CPC-PHBV construct (Xue et al., 2022[9]). However, after careful evaluation of the research-based investigations published regarding the treatment of OA, multimodal approaches must be applied to regenerate or repair the articular cartilage satisfactorily. Notably, several molecular key players like bone morphogenic proteins (BMPs) and Wnt/β-catenin signaling pathways were found essential to maintain the homeostasis between articular cartilage and endochondral ossification and are involved both in normal functioning and in pathogenesis of OA (Chawla et al., 2022[1]; Jaswal et al., 2023[2]). Thus, their modulation seems essential to effectively target and treat OA. To achieve these from a Bioglass platform, a precise customization of former is necessary. Covalently linking the different molecular signaling pathways agonist/antagonist that are capable for promoting the articular cartilage repair or growth; along with cartilage regeneration promoters, such as chondrocytes transforming bone marrow stromal cells (BMSCs) embedding, and inflammatory pathway inhibitors to Bioglass and then using it as a single platform (scaffold) can be the ideal approach. Moreover, as the field of Bioglass would progress further, it shall be necessary to optimize these novel biomaterials, since it is essential to possess a comprehensive knowledge of their inherent structures and characteristics. Next, the imaging and quantification of articular cartilage regeneration or repair may currently be achieved using non-destructive techniques such as μCT imaging and histopathological analysis (Jones et al., 2010[4]; Yue et al., 2011[10]); and their therapeutic abilities can be correlated and confirmed through the biological response observed in *in vivo* settings.

## Notes

Keshav Raj Paudel and Amitabha Bandyopadhyay (Department of Biological Sciences and Bioengineering (BSBE), Indian Institute of Technology, Kanpur, 208016, Uttar Pradesh, India; E-mail: abandopa@iitk.ac.in) contributed equally as corresponding author.

## Declaration

### Conflict of interest

The authors have no conflict of interests to declare.

### Funding

None.
